# Blood Lead Levels and Associated Sociodemographic Factors among Children Aged 3 to 14 Years Living near Zinc and Lead Mines in Two Provinces in Vietnam

**DOI:** 10.1155/2021/5597867

**Published:** 2021-07-06

**Authors:** Thi Giang Hoang, Quang Phuc Tran, Van Tung Lo, Ngoc Hai Doan, Thu Ha Nguyen, Minh Khue Pham

**Affiliations:** ^1^Faculty of Public Health, Haiphong University of Medicine and Pharmacy, Vietnam; ^2^National Institute of Occupational and Environmental Health, Ministry of Health, Vietnam

## Abstract

Lead poisoning in children is a major public health concern worldwide, especially in developing countries. We conducted a cross-sectional study on 403 children aged from 3 to 14 years living nearly zinc–lead mining areas in two provinces in Vietnam (Bac Kan and Thai Nguyen) from 06/2016 to 10/2016 to identify risk factors for lead contamination. *Results*. The proportion of children with blood lead levels (BLLs) ≥ 10 *μ*g/dL was 80.51% in Bac Kan and 50% in Thai Nguyen; the mean blood lead level for children was 14.41 ± 9.42 *μ*g/dL. In linear regression analyses, the body mass index was negatively associated with elevated BLLs with *r* = −0.404, *p* < 0.05 (95% CI: -0.801, -0.006). In multivariable regression analysis, several risk factors were associated with lead contamination including male sex (aOR = 2.44, 95% CI: 1.13-5.24, *p* = 0.02), play areas in Bac Kan (aOR = 2.3 (1.02-5.17), *p* = 0.04), proximity of children's home of less than 2 kilometers from the mine (aOR = 2.90 (1.54-5.44), *p* = 0.001), and inattentive symptoms in Thai Nguyen (aOR = 7.85, 95% CI 3.49-17.69, *p* = 0.001). Environmental factors, including lead concentrations in the soil and ambient air samples in both locations, are many times higher than Vietnamese standards.

## 1. Introduction

Childhood lead poisoning is a major public health concern worldwide. The highest burden is in the low- and middle-income countries [1]. When children are exposed to lead, even at low levels of exposure, there are harmful effects on their mental and physical development, impairing their health and intelligence, and can significantly affect their families as well as society [2–6].

The Institute for Health Metrics and Evaluation (IHME) estimated that in 2017, lead exposure accounted for 1.06 million deaths and 24.4 million years of healthy life lost (disability-adjusted life years (DALYs)) worldwide. IHME also estimated that in 2016, lead exposure accounted for 63.2% of the global burden of idiopathic developmental intellectual disability, 10.3% of the global burden of hypertensive heart disease, 5.6% of the global burden of ischaemic heart disease, and 6.2% of the global burden of stroke [7].

According to the CDC, human blood lead levels (BLLs) are considered a warning when they exceed a threshold of 10 *μ*g/dL [8]. For elevated BLLs, chelatin treatment should be considered when BLLs is above 45 *μ*g/dL for children and above 70 *μ*g/dL for adults. As several recent research results have shown concerns about lower exposure levels in children, the CDC has introduced a new safe reference threshold for BLLs below 5 *μ*g/dL in children [9].

Several studies have shown that BLLs above 10 *μ*g/dL in children were associated with age, gender, race/ethnicity, use of folk medicine, cosmetics, age of housing, living near lead-polluted areas, parental occupation, passive smoking in the family, having a sibling, living in a crowded neighborhood, and drinking from a water source [6, 10–13].

Lead poisoning disease has been recognized as an occupational disease in Vietnam since 1998 and is therefore covered under the social insurance system. Several lead poisoning prevention measures have also been implemented, particularly focused on pollution-risk areas [1, 14, 15]. For instance, leaded gasoline has been completely banned since 2001. However, Vietnamese children still face the risk of lead contamination from many sources such as activities in craft villages, mining areas, toys, food, and in some traditional medicines. In 2015, a study was conducted on 186 children aged 0-5 years old in the lead recycling village of Dong Mai, Hung Yen, and showed 70.4% of the children were poisoned at mild levels (BLLs from 10 to 45 *μ*g/dL) [16].

Thai Nguyen and Bac Kan are provinces located in the North East Vietnam with many lead-zinc mining that have existed for decades. They represent the main driving force for economic development but also cause serious lead pollution problems in the regions. Despite the local authority's efforts to reduce and prevent lead pollution, these mines with outdated technology and limited resources still exist. Research in Tan Long, Thai Nguyen, showed that lead concentrations in lead-zinc mines exceed the permitted standards by 186 times, while the agricultural and forestry soil samples exceed the permitted standards by 1.6 to 5.8 times [17]. In Bac Kan, a study at the Cho Dien zinc–lead mine area showed that 100% of soil samples and 45.4% of drinking water samples exceeded the permitted standards for lead [18].

The problem of lead poisoning for workers and people living near these areas is of great concern. However, there is a lack of research on lead contamination in children and associated factors. Given these concerns, the objective of this study was to (1) determine the prevalence of lead poisoning among children ages 3 to 14 years old living near mining at Tan Long, Thai Nguyen and Ban Thi, Bac Kan and (2) assess the risk factors associated with high BLLs in this population.

## 2. Material and Methods

The Strengthening the Reporting of Observational Studies in Epidemiology (STROBE) statement guidelines was followed for reporting this cross-sectional study.

### 2.1. Study Design

The study was a part of a national—level research project in Vietnam conducted in two phases. This is the first phase of the project and uses a cross-sectional study design. Based on previous studies in China [13], we estimated 15% of children would have a BLL ≥ 10 *μ*g/dL (there were no data in this population in Vietnam), and the sample size required for the blood lead survey was approximately 195 children at each study site, which would provide a margin of error around the prevalence estimate with 95% CI [19]. The study population included children who lived in a 10-kilometer radius around zinc–lead mining in Bac Kan and Thai Nguyen provinces (the areas most affected by mining activities).

### 2.2. Setting

**Figure 1:**
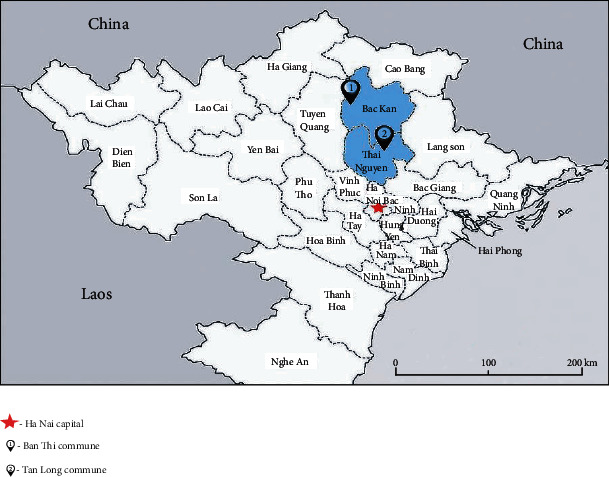
Map of Bac Kan and Thai Nguyen provinces.

The study was conducted from June to October 2016 in two communes where largest zinc–lead mining sites exist, in Bac Kan (Ban Thi Commune) and Thai Nguyen (Tan Long Commune) (Figure 1).

**Figure 2:**
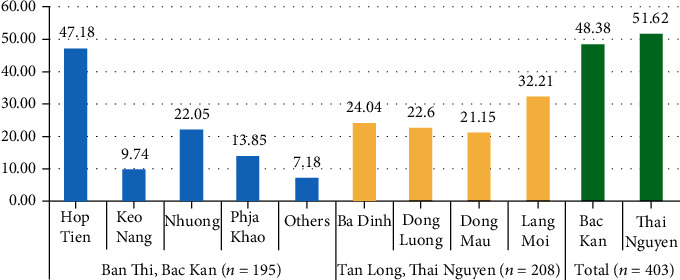
Distribution of study population according to setting.

Ban Thi Commune is located in the Cho Don district and covers an area of 65 square kilometers with a population of approximately 1500 people. There are 8 villages in Ban Thi Commune, and all were selected for the study: Phia Khao, Ban Nhuong, Hop Tien, Keo Nang, Tham Tau, Phieng Lam, Ban Nhai, and Khuoi ken. Tan Long Commune is located in Dong Hy district, covers an area of 48 square kilometers, and has a population of approximately 5200 people. There are 9 villages in this commune; 4 villages were selected including Ba Dinh, Dong Luong, Lang Mau, and Lang Moi. The villages were contaminated by hydrogeological and geochemical lead–zinc mines. The number of children in each village is given in Figure 2.

### 2.3. Participants

We enrolled 403 children ages 3–14 years old into the study, including 195 children in Ban Thi Commune and 208 children in Tan Long Commune. The eligibility criteria included the following:

- Age of 3 (36 months) to 14 years old
- Born and grew up in one of the 12 villages cited above
- Do not suffer from serious diseases such as cerebral palsy and disability (cannot move)

A simple random sample was used to select and enroll a minimum of 195 participants at each study site from the list of 275 eligible children in Ban Thi and 305 eligible children in Tan Long. All children were accompanied to the study site (local medical station) by parents or legal guardians. They were all informed and signed a consent form for participating in the survey on behalf of each child before the sampling, and interviews were conducted.

The distribution of children by province, gender, and age group is presented in Table 1.

### 2.4. Measurements

#### 2.4.1. Blood Collection and Analysis

A blood volume of 1.52 mL was taken from each child. Blood sampling was done at the Commune Health Station. To prevent external contamination, the venipuncture site was meticulously cleaned by alcohol cotton. Each blood sample was collected in an EDTA tube and transported to the National Institute of Occupational and Environment Health (NIOEH) laboratory in dry ice packs. In the laboratory, samples were stored in a refrigerator at –20°C until analyzed. Blood samples were frozen until reaching room temperature and diluted at 1 : 25 in the diluent solution (0.2% (*v*/*v*) nitric acid, 0.05% *w*/*v* Triton-X-100). After dilution, the samples were mixed well by vortex and centrifuge. Lead concentration in blood was measured by inductively coupled plasma-mass spectrometry (ICP/MS NexION 350X-Perkin Elmer; LOD: 0.8 *μ*g/dL; recovery: 89.7-101.2% (ICP/MSELAN900-Perkin Elmer); detection limit of Pb is 0.0001 mg/L) [20]. Besides BLL testing, haemoglobin (Hb) was measured at the NIOEH's lab as well.

#### 2.4.2. Sociodemographic Information

Sociodemographic information obtained from the questionnaire included sex, age, parental educations, and occupation (unemployed, employed at the lead–zinc mine, and other jobs), housing type, proximity to the mine, drinking water source, parental smoking at home, antecedents of using traditional medicine (containing lead derivatives–*Thuốc cam*), usual play areas for children, and hand washing before meals of children. Parents filled out the sociodemographic questionnaire during their meeting at the medical station where blood samples and examinations (height, weight measerument) were conducted on each child.

#### 2.4.3. Body Mass Index (BMI) Measurement

Height and weight were measured mechanical weight and height scale (RGZ–120) before collecting the blood samples with an accuracy of 0.1 cm in height and 0.1 kg in weight.

#### 2.4.4. The Assessment of Children's Attention Deficit Hyperactivity Disorder (ADHD) Symptoms and Comorbidities by Using the Vanderbilt ADHD Diagnostic Parent Rating Scale (VADPRS)

VADPRS assessed symptoms including inattentive (items 1–9), hyperactivity/impulsivity (items 10–18) symptoms, and 2 comorbidities (conduct/oppositional defiant disorders, items 19–40 and anxiety/depression, (items 41–47). The VADPRS is developed based on the DSM-V ADHD diagnostic criteria to help pediatricians identify which children are at risk or not at risk for the diagnosis of ADHD symptoms and comorbidities [21, 22] and is widely used in school or preschool aged children [23, 24]. All symptoms were rated on a 4-point scale that indicated how frequently each symptom occurred. Parents were interviewed on their children's behavior during the past 6 months by a trained interviewer at the medical station.

#### 2.4.5. Soil Sampling

Residential soil samples were taken at a depth of 0.5-1.0 cm at 30 different locations (in the home garden and along the road). Each soil sample taken was approximately 300 g and was preserved in polyethylene bags. Samples were dried to a constant weight at 45°C, mashed, blended, and sieved with a 2 mm sieve.

#### 2.4.6. Sampling Ambient Air

Air sampling was collected by Kimoto equipment (Japan), Gelman absorbed tuber MCE filter (45 *μ*m, *d* = 37 mm).

#### 2.4.7. Sampling Drinking Water

Samples of drinking water were taken from the household taps. The volume of each water sample was 500 mL and was stored in a clean polyethylene bottle and stored at room temperature until analysis.

All samples were collected and stored and transported to the NIOEH Lab. Lead concentrations in soil, ambient air, and drinking water were analyzed by inductively coupled plasma-mass spectrometry (ICP/MS ELAN900-PerkinElmer) [20].

All investigators were carefully trained before implementing the study to minimize bias.

### 2.5. Statistical Analysis

Data were entered and cleaned using EPIDATA 3.1 software and analyzed using STATA 14.0 software. Means with standard deviation (SD), minimum and maximum values were calculated to describe the BLL distributions. BLLs were then classified into the following categories: <5 *μ*g/dL, 5- <10 *μ*g/dL, 10–45 *μ*g/dL, and >45 *μ*g/dL. The categories of elevated BLLs (above 10 *μ*g/dL) were compared among groups based on sociodemographic characteristics. Since BLLs were not normally distributed, one-way nonparametric analyses of variance (Wilcoxon or Kruskal-Wallis test) was used to compare the mean of BLLs. Pearson's correlation was used for assessing the correlation between BLLs and independent variables. Bivariate and multivariable logistic regression analyses were performed to explore the associations between the independent factors and BLL above 10 *μ*g/dL (≥10 *μ*g/dL vs. <10 *μ*g/dL) and above 5 *μ*g/dL (≥5 *μ*g/dL vs. <5 *μ*g/dL). All variables with a *p* value below 0.2 in the bivariate analysis were included in the multivariable analysis, and the goodness-of-fit test was used to consider the suitability of the model by using McFadden's pseudo *R*^2^ indicator. A probability level of 0.05 or less was considered significant.

### 2.6. Ethical Consideration

The protocol research was approved by the Institutional Ethical Committees of the Vietnam National Institute of Occupational and Environmental Health (coded DTDLCN-48/15/01). The parents or legal guardians were all informed and signed informed consents for participating in the survey on behalf of the child before the sampling, and interviews were conducted.

## 3. Results

### 3.1. Prevalence of Elevated Blood Lead Levels

Overall, 64.7% of the children had an elevated BLLs (above 10 *μ*g/dL); the mean ± standard deviation (SD) of BLLs was 14.41 ± 9.42 *μ*g/dL (95% CI: 13.48–15.33 *μ*g/dL). Both the proportion of children with elevated BLLs and the mean of BLLs were significantly higher in Ban Thi, Bac Kan commune compared to Tan Long, Thai Nguyen (80.5% vs. 50.0% and 15.42 ± 6.45 *μ*g/dL vs. 13.47 ± 11.48 *μ*g/dL, respectively, *p* < 0.001 for both comparisons). There were 4 children (1.9%) in Tan Long commune that had BLLs above 45 *μ*g/dL (Table 2).

In Ban Thi, Bac Kan, the mean ± SD of BLLs was highest in children aged <6 years and was lowest in children aged 11-14 years (16.9 ± 6.74 vs. 13.92 ± 5.58 *μ*g/dL, *p* = 0.08); there were significant differences in BLLs when comparing boys and girls (16.45 ± 5.98 vs. 14.06 ± 6.80 *μ*g/dL, *p* < 0.001). In Tan Long, no significant association was observed between BLLs and age group, or sex (Table 3).

### 3.2. Risk Factors Associated with Elevated BLLs

In linear regression analyses, 6 factors were selected to identify correlations with BLLs (*μ*g/dL) including age (years), height (cm), weight (kg), chest index (cm), BMI (kg/m^2^), and haemoglobin (g/L). We identified 3 negative correlations with BLLs, including height, weight, and BMI in Ban Thi, Bac Kan (*p* < 0.05). However, in regression diagnostics using variance inflation factors for indepentdent variables (vif), a multicollinearity relationship was detected between height, weight, chest index, and BMI (vif > 10). We therefore obtain these 3 factors from the model. The final model showed BLLs inversely and significantly correlated with a decreasing BMI in Ban Thi, Bac Kan, with *r* = −0.404, *p* < 0.05 (95% CI: -0.801, -0.006) (vif < 2). No significant association was identified between BLLs and age, BMI, and haemoglobin in Tan Long (Table 4).

Logistic regression analyses were performed to identify associations between elevated BLLs (>10 *μ*g/dL) and categorical factors for each site including gender, age, antecedent of traditional medicine, hand washing before meals, housing type, play areas, close proximity of house to the mine, drinking water source, parental job, parental education degree, and Vanderbilt ADHD Diagnostic Parent Rating Scale. As shown in Table 5, in Ban Thi, two association factors were observed with high BLLs. These include gender and play areas for children. Compared with girls, boys were more likely to have BLLs above 10 *μ*g/dL (aOR = 2.44, 95% CI 1.13-5.24, *p* < 0.05). The children who play usually on the ground had more than twice the odds ratio of having BLLs above 10 *μ*g/dL (aOR = 2.3, 95% CI 1.02-5.15, *p* < 0.05). None of the parent's characteristics were associated with elevated BLLs. In Tan Long, findings were somewhat different compared to Ban Thi. The close proximity of the children's house to the lead–zinc mine (less than 2 kilometers) increased the risk of having BLLs above 10 *μ*g/dL by 2.9 (aOR = 2.90, 95% CI 1.99-5.44, *p* < 0.05). Presenting inattentive by VADPRS was strongly associated with the prevalence of BLL above 10 *μ*g/dL (aOR = 7.85, 95% CI 3.49-17.69, *p* < 0.01). Neither parents'characteristics nor children's sociodemographics were associated with elevated BLLs (Table 5).

The concentration of lead in the soil samples in Ban Thi, BK, was 10 times higher than in Tan Long, TN, and nearly 5000 times higher than the permitted standard in Vietnam. All of the ambient air samples also exceed the Vietnamese permitted standards. The average concentration of lead in drinking water in the two study sites did not exceed the permitted standards in Vietnam (although approximately 10% of the samples exceeded the permitted standards) (Table 6).

## 4. Discussion

Our study identified a high prevalence of lead poisoning in two sites near lead–zinc mines, Ban Thi and Tan Long. According to the CDC US recommendation, childhood BLLs should be maintained below 5 *μ*g/dL; we found that up to 89% of the children at the study site were exposed to levels that exceeded this. The present study showed that lead can affect mental intellectual development even at very low levels of exposure [3–5]. Effects include impaired cognitive, motor, and behavioral abilities such as having attention deficit hyperactivity disorder [6]. We also found that the mean ± standard deviation of BLLs in children was 15.42 ± 6.45 *μ*g/dL (95% CI 14.5-16.33) in Ban Thi and 13.47 ± 11.48 *μ*g/dL (95% CI 11.9–15.04) in Tan Long, which were levels 2 to 3 times the CDC recommendation. We also noted that 4 of the 208 children had BLLs above 45 *μ*g/dL, the threshold for initiating chelation therapy.

A similar phenomenon has been reported in other mining areas. In Nigeria, 59% (204/345) of children < 5 years old experienced lead poisoning (≥10 *μ*g/dL) and 97% (198/204) of children had blood lead levels (BLLs) ≥ 45 *μ*g/dL (gold ore was processed in two-thirds of the family compounds surveyed) [25, 26].

Another important finding was the association between high BLLs in children with low BMI and attention deficit manifestations. As this study was a cross-sectional design, we could not assess causality between the BLLs and these factors, but these findings may indicate that lead contamination is related to a compromised nutritional and mental status. The direct relationship between elevated BLLs and nutritional status has been shown in other studies. Poor nutritional status such as deficiencies in calcium and iron can increase the body's capacity for absorption of lead and therefore may lead individuals to be more prone to having high levels of lead in their blood [27]. Lead poisoning can also affect somatic growth and mental health development in children [6, 28]. In this study, we found that Hb values did correlate negatively with high BLLs but the relationship was not statistically significant.

Other risk factors for lead poisoning identified in our study included gender, play areas, and the proximity of children's house to the mines. The relationship between gender and high BLLs has been shown in many studies [12, 29, 30]. Boys spend more time doing outdoor activities compared to girls and might have more hand-to-mouth and object-to-mouth activities, which increase the risk of lead contamination [31]. Lead can be absorbed into the body, causing poisoning through all three pathways of respiratory, digestive, and through skin and mucous membranes. For children, the risk of lead entering the body is mainly through the gastrointestinal tract due to children's habit of sucking objects, toys, or playing on dirty floors and poor hand hygiene. Therefore, lead poisoning in children is also known as “hand-to-mouth” disease and children's hand hygiene is one of the key points in lead poisoning prevention recommendations for children. In our study, we did not collect information on hand-to-mouth activities, so the factors associated with our observed gender differences can only be postulated. Our research results did not find an association between the rate of elevated BLLs and the child's history of using folk medicine (*Thuốc cam*), frequency of hand washing before meals, or drinking water sources used in the family. However, we did note regional characteristics. Play areas on soil and a distance from home to mines of less than 2 km are factors associated with the risk of lead poisoning in children. These results are in line with results of environmental measurements, when lead concentration in the soil and ambient air exceed the Vietnamese permitted standards. In observing the local culture, we saw that in addition to having lead and zinc mines, some citizens also stored ore slag or took advantage of the mines' land to build yards and houses, further increasing the children's risks of lead poisoning.

As recommended by WHO [2], children are a vulnerable population for lead poisoning due to many environmental risk factors including leaded gasoline, mining activities, leaded paint and pigments, lead-contaminated food, waste with lead from incineration, ceramic glaze, lead-contaminated water, some herbs, folk medicine, cosmetics, and toys [16, 26, 32, 33]. The environmental factors assessed in this study included soil, ambient air, and drinking water. Average lead concentration in residential soil in Ban Thi, BK, was 10 times higher than that in Tan Long, TN, and nearly 5000 times higher than the permitted standard in Vietnam. All of the samples exceeded the permitted standard for lead concentration in the ambient air. With respect to pollution in drinking water, the results showed that approximately 10% of samples have lead concentrations exceeding the permitted standards in Vietnam. The problem of lead pollution in the environment has been known for many years despite intervention by the local authorities [15, 17, 18]. Typically, research conducted by author Nguyen Thi Thu Hien (2012) showed that the lead concentration in soil and drinking water in 3 villages in Ban Thi (Phja Khao, Ban Nhuong, and Hop Tien) was much lower than our study. This may indicate that the accumulation of lead in the soil over time may be due to mining and production activities [18]. These findings show an alarming risk of lead contamination in children and the urgent need to have interventions for these environmental issues in these specific residential areas, in parallel with medical interventions.

One of the limitations of the study was that with the limited sources, only 60 environmental samples were taken, so we could not measure the relation of lead in the environment with BLLs of children, as well as some other risk factors such as toys, dust, and paint. Thus, the goodness-of-fit indicators on the final logistic regression models were rather small, which indicated that the number of variables included in the model might be not enough. However, the associated factors found in this study, including male sex, play areas in soil, closely residence to the mines, and inattentive syndrome were mentioned in the previous research [3, 12, 34, 35], may also help to support the validity of our findings. Therefore, further study is needed to be able to understand the lead poisoning issues in these settings.

## 5. Conclusions

Childhood lead poisoning is a great concern in mining areas despite lead gasoline being phased out in the country and the effort of local authorities to control lead pollution caused by the mines. Based on the findings of this study, interventions focused on relocating households away from contaminated areas are needed in order to address this situation.

## Figures and Tables

**Table 1:** Distribution of study population by gender and age.

		Ban Thi, Bac Kan (*n* = 195)		Tan long, Thai Nguyen (*n* = 208)		Total (*n* = 403)	
		*n*	%	*n*	%	*n*	%
Gender	Boys	110	56.4	123	59.1	233	57.8
	Girls	85	43.6	85	40.9	170	42.2

Age group	<6	45	23.0	55	26.5	100	24.8
	6-10	113	58.0	97	46.6	210	52.1
	11-14	37	19.0	56	26.9	93	23.1
	Mean (SD)	8.61 ± 2.90		8.17 ± 2.88		8.40 ± 3.15	

**Table 2:** Blood lead levels in children in Ban Thi and Tan Long communes.

BLLs (*μ*g/dL)	Ban Thi, bac Kan^1^ (*n* = 195)	Tan long, Thai Nguyen^2^ (*n* = 208)	*p* value ^(1 vs. 2)^	Total (*n* = 403)
<5	1 (0.5)	45 (21.6)	<0.001^∗^	46 (11.4)
5-<10	37 (19.0)	55 (26.5)		92 (22.8)
≥10–45	157 (80.5)	104 (50.0)		261 (64.7)
>45	0 (0)	4 (1.9)		4 (1.0)
Mean ± SD (95% CI)	15.42 ± 6.45 (14.5-16.33)	13.47 ± 11.48 (11.9–15.04)	<0.001^∗∗^	14.41 ± 9.42 (13.48–15.33)
Min-max	4.97–37.99	0.1–61.49		0.1–61.49

^∗^Fisher exact test. ^∗∗^Mann–Whitney test.

**Table 3:** Differences in blood lead levels (*μ*g/dL) by gender and age groups.

		Ban Thi, Bac Kan			Tan Long, Thai Nguyen		
		*n*	Mean ± SD	95% CI	*n*	Mean ± SD	95% CI
Age group (years)	<6	45	16.9 ± 6.74	14.87-18.92	55	12.94 ± 11.11	9.93-15.94
	6-10	113	15.31 ± 6.52	14.09-16.52	97	13.31 ± 11.28	11.03-15.57
	11-14	37	13.92 ± 5.58	12.05-15.77	56	14.29 ± 12.33	10.98-17.58

*p* value^∗^			0.08			0.78	

Gender	Boys	110	16.45 ± 5.98	15.32-17.59	123	13.84 ± 11.19	11.84-15.84
	Girls	85	14.06 ± 6.80	12.59-15.52	85	12.92 ± 11.92	10.35-15.5

*p* value^∗∗^			<0.001			0.36	

^∗^Kruskal-Wallis test. ^∗∗^Mann–Whitney test.

**Table 4:** Correlation of blood lead levels with age, BMI, and haemoglobin.

Parameter	Ban Thi, Bac Kan		Tan Long, Thai Nguyen	
	Coefficient 95% CI	*p* value	Coefficient 95% CI	*p* value
Age group (years)	0.015 (-0.328, 0.360)	0.92	0.106 (-0.476, 0.688)	0.72
BMI	-0.404 (-0.801, -0.006)	0.04	-0.152 (-0.989, 0.684)	0.72
Haemoglobin (g/L)	-0.079 (-1.174, 0.016)	0.10	-0.036 (-0.190, 0.117)	0.64

**Table 5:** Logistic regression analysis of blood lead levels above 10 (*μ*g/dL) among children in Ban Thi and Tan Long Communes.

Characteristics		Ban Thi-Bac Kan		Tan Long-Thai Nguyen	
		OR (95% CI)	Adjust OR (95% CI)^a^	OR (95% CI)	Adjust OR (95% CI)^b^
Gender	Boys (vs. girls)	2.34 (1.13-4.85)^∗^	2.44 (1.13–5.24)^∗^	1.18 (0.68–2.06)	

Age	<6 (vs. 11-14)	2.20 (0.65-7.44)	2.16 (0.58-7.98)	0.96 (0.45–2.03)	
	6-10 (vs. 11-14)	0.97 (0.39-2.38)	0.96 (0.37-2.50)	0.88 (0.45–1.70)	

Ever using “thuốc cam”	Yes	0.87 (0.23–3.31)		1.15 (0.58–2.27)	

Hand washing before meals	Yes	1.1 (0.42-2.9)		0.93 (0.54-1.61)	

Housing type	Stilt house (vs. modern house)	1.52 (0.73–3.18)		1.25 (0.72–2.18)	

Play areas	Soil (vs. brick)	2.18 (1.01-4.71)^∗^	2.3 (1.02-5.17)^∗^	0.99 (0.57-1.75)	

Close proximity of house to the mine (≤2 km)	Yes	1.01 (0.36-3.26)		2.58 (1.45-4.58)^∗∗^	2.90 (1.54-5.44)^∗∗^

Drinking water source	Not treated water (vs. treated water)	1.07 (0.52-2.20)		1.39 (0.80-2.42)	

Parent working at the mine	Yes (vs. no)	0.65 (0.30-1.41)		0.97 (0.45-2.11)	

Mother's highest degree	Primary school or less (vs. high school/college/university)	1.94 (0.22-16.74)		0.62 (0.18-2.09)	
	Middle school (vs. high school/college/university)	1.27 (0.61-2.62)		0.93 (0.53-1.64)	

Father's highest degree	Primary school or less (vs. high school/college/university)	1.16 (0.23-5.80)		1.18 (0.25-5.51)	
	Middle school (vs. high school/college/university)	0.90 (0.43-1.88)		0.88 (0.50-1.54)	

Vanderbilt ADHD Diagnostic Parent Rating Scale	Inattentive (yes vs. no)	0.60 (0.26-1.39)		7.22 (3.22-15.82)^∗∗^	7.85 (3.49-17.69)^∗∗^
	Hyperactive-impulsive (yes vs. no)	0.64 (0.25-1.66)		1.11 (0.33-3.78)	
	Conduct/oppositional defiant disorder (yes vs. no)	0.23 (0.03-1.70)	0.29 (0.03-2.33)	0.9 (0.05-14.99)	
	Anxiety/depression (yes vs. no)	0.43 (0.18-1.02)	0.54 (0.21-1.38)	1.31 (0.40-4.29)	

^∗^ *p* value < 0.05. ^∗∗^*p* value < 0.01. ^a^McFadden's pseudo *R*^2^ = 0.086. ^b^McFadden's pseudo *R*^2^ = 0.148.

**Table 6:** Lead in soils, ambient air, and drinking water samples.

Sample	Study site	*N*	Average ± SE	Min-max	Exceed VN standard^∗^ (*n*, %)
Soils (mg/kg) (VN standards ≤ 70)^∗^	Ban Thi (BK)	30	2980.23 ± 1112.96	0.4-33820.62	27 (90.0)
	Tan Long (TN)	30	263.46 ± 67.15	11.72-1790.36	22 (73.33)

Ambient air (*μ*g/m^3^) (VN standards ≤ 1.5)^∗^	Ban Thi (BK)	30	5.89 ± 0.76	1.6-18.5	30 (100.0)
	Tan Long (TN)	30	6.79 ± 0.98	2-30.2	30 (100.0)

Drinking water (mg/L) (VN standards ≤ 0.01)^∗^	Ban Thi (BK)	31	0.0033 ± 0.0005	0.002-0.0135	3 (9.68)
	Tan Long (TN)	30	0.0077 ± 0.0034	0.0002-0.0994	3 (13.33)

^∗^Vietnamese standards.

## Data Availability

The EXCEL/STATA data used to support the findings of this study are available from the corresponding author upon request.

## Abbreviations

ADHD: Attention deficit hyperactivity disorder
VADPRS: Vanderbilt ADHD Diagnostic Parent Rating Scale
BK: Bac Kan
BLL: Blood lead level
BMI: Body mass index
CDC US: United States Centers for Disease Control and Prevention
ICP/MS: Inductively coupled plasma-mass spectrometry
IHME: Institute for Health Metrics and Evaluation
MOH: Ministry of Health
NIOEH: Vietnam National Institute of Occupational and Environmental Health
TN: Thai Nguyen
VN: Vietnam
WHO: World Health Organization.
